# The Story of the Insane from Year to Year

**Published:** 1892-02-06

**Authors:** 


### The Story of the Insane from Year to Year.

(Fourth Series).

### NORFOLK COUNTY ASYLUM.

On December 31st, 1890, there were in this asylum 687 patients, being a decrease of one on the numbers for the previous year. The admissions were 165, the recoveries 81, and the deaths 76. The percentage of recoveries was very high —namely, 52 59—and the death-rate was 10 65, just a shade above the average. The Norfolk people would seem to be a sober lot, comparatively at least, for only 9 of the total admissions were caused by drink, but 57 were owing to hereditary influence. Of the 76 deaths 33 were caused by various cerebral diseases, and 11 by phthisis. There were 2 from typhoid, and 2 from dysentery. As usual, Dr. Thomson's report is one of the clearest and best arranged we get to review. It shows a thorough grasp of the asylum in all its details, and bears evidence of much energy and ability in management. Referring to the recoveries and to the silly proposal to build an acute hospital in London, Dr. Thomson says: "The belief that insanity is a malady not often recovered from is not confined to the non-medical public, for a committee of the London County Council, several of whom are medical men, under the impression that little or nothing was being done for the insane in existing asylums, proposed to erect a hospital for the insane in London. This scheme, I am glad to say, would not bear investigation, and was abandoned. Asylum recovery rates compare favourably with ordinary hospital recovery rates; but, unfortunately, the asylums cannot turn out their incurables as hospitals can, hence the accumulation of insane patients, and the outcry about the increase of asylums and insanity." It would be interesting to have heard what Dr. Thomson thinks of the New Lunacy Act. He only tells us that the year 1890 will be long remembered in asylums as one marked by many medico-legal and financial innovations caused by the new Local Government and Lunacy Acts. The Visiting Committee say they "have pleasure in again recording their satisfaction with the efficient manner in which Dr. Thomson has managed the establishment during the past year." There is a balance of £162 in favour of the farm, and rent of land is charged in the accounts. The weekly rate of maintenance is 9s. 8§d. per head.

### DERBY COUNTY ASYLUM.

The numbers in this asylum rose during the year from 430 to 448. There were 160 admissions, 66 recoveries, and 51 deaths. The recoveries reach the creditable per centage of 44·2, and the deaths work out at 11·4. Intemperance in drink accounts for 14 of the admissions, and hereditary influence for 54. Of the deaths, 25 were caused by brain diseases, and 14 by consumption. Dr. Lindsay says that "the question of making suitable provision for young idiots and imbeciles having been brought before the County Council by Mr. H. Wardle, M.P., was referred to the Asylum Committee for re-consideration, and reported upon by them to the County Council at their meeting on January 14th, when a motion was passed to appoint a small committee to consult with other County Councils in the Midland districts as to the advisability of making joint provision for idiots and imbeciles under the age of 18 years." We hope that something may come of this reporting and counter-reporting, and that Dr. Lindsay will be able to tell us that the various counties have agreed on something definite before the Greek Kalends. The attendants' leave of absence has been increased, which seems a step in the right direction. We hope Dr. Lindsay may not have the same experience as Dr. Davies, of Kent, had respecting this extra leave. One of the provisions of the New Lunacy Act related to the assessment of asylums, but, like so much else in the Act, it was ill-considered, and no basis of rating was stated. The result, of course, was that large sums of money were wasted in valuations, and even then great discrepancies exist. Dr. Lindsay points this flaw out with his usual clearness of diction. "For example," he says, "this asylum, with accommodation for 471 patients, is assessed on the basis of £3,000, whereas another asylum with 490 beds is assessed at £1,000, less than half the amount of the smaller asylum; and a third asylum with 1,200 beds, over two and a-half times the size of this asylum, is assessed at the same rate, £3,000." Parallel cases might easily be found, and all these anomalies might easily have been avoided, a fixed sum per bed-space been named, such space to be reckoned on the space required by the printed instructions of the Commissioners in Lunacy. Indeed, the more one examines this wretched Act the worse it seems. Dr. Lindsay adds that the New Lunacy Act "has thrown an increased and steadily increasing amount of work upon the medical officers." Owing to the delay caused by the Government audit the accounts are not published.

### LANCASHIRE COUNTY ASYLUM AT PRESTWICH.

Not fewer than 2,314 patients were in residence here at the beginning of the year 1890, and this number had increased to 2,327 on the first day of 1891. The admissions were 841; the recoveries 291, and the deaths 287. Intemperance in drink was the cause of insanity in 163 cases, and hereditary influence in 186. Of the deaths, 105 were owing to cerebral or spinal diseases, and 58 to phthisis. We are in full accord with Mr. Ley when he says that "the policy which dictated in the past the establishment of lunatic wards in connection with workhouses and the transfer thereto of chronic and incurable cases from the asylum has been of questionable benefit to the insane." When he criticises the new Lunacy Act every word of the paragraph may be laid to heart. "We have now," proceeds Mr. Ley, "had nearly a year's experience of the working of the new Lunacy Act, and those who looked with disfavour upon the Bill before its passing have had their prognostications verified since the Act has been in operation. Viewed from every standpoint, the Act is a failure; instead of conferring benefit upon the patient, it erects a powerful barrier against his treatment, without giving society any additional protection or safeguard. . . . There was enough and to spare of red tape in asylums before, and registers, returns, and case books were unnecessarily numerous; but the present Act and the Lunacy Commissioners' orders build up bulwarks of pasteboard, add considerably to the statutory duties of the officials, and impose upon the Superintendent a large amount of clerical work, which, if done by him, must be done to the exclusion of other and more important duties bearing upon the treatment and welfare of the patients." We heartily join with the Visiting Committee in their expressions of pleasure at the recovery of "their highly-esteemed medical Superintendent," who had for some time been suffering from the effects of an assault. There is a balance of £636 in favour of the farms obtained in the manner which seems customary in asylums. The weekly cost per head was 8s. 3§d.

